# A falciform ligament flap surface sealing technique for laparoscopic and robotic-assisted liver surgery

**DOI:** 10.1038/s41598-020-69211-8

**Published:** 2020-07-22

**Authors:** M. Rahimli, A. Perrakis, V. Schellerer, M. Andric, J. Stockheim, E. Lorenz, M. Franz, J. Arend, R. S. Croner

**Affiliations:** 1 Department of General, Visceral, Vascular and Transplant Surgery, University Hospital Magdeburg, Leipziger Str. 44, 39120 Magdeburg, Germany; 2 Department of Pediatric Surgery, University Hospital Erlangen, Krankenhausstraße 12, 91054 Erlangen, Germany

**Keywords:** Medical research, Outcomes research

## Abstract

Whether sealing the hepatic resection surface after liver surgery decreases morbidity is still unclear. Nevertheless, various methods and materials are currently in use for this procedure. Here, we describe our experience with a simple technique using a mobilized falciform ligament flap in minimally invasive liver surgery (MILS). We retrospectively analyzed the charts from 46 patients who received minor MILS between 2011 and 2019 from the same surgical team in a university hospital setting in Germany. Twenty-four patients underwent laparoscopic liver resection, and 22 patients received robotic-assisted liver resection. Sixteen patients in the laparoscopic group and fourteen in the robotic group received a falciform ligament flap (FLF) to cover the resection surface after liver surgery. Our cohort was thus divided into two groups: laparoscopic and robotic patients with (MILS + FLF) and without an FLF (MILS−FLF). Twenty-eight patients (60.9%) in our cohort were male. The overall mean age was 56.8 years (SD 16.8). The mean operating time was 249 min in the MILS + FLF group vs. 235 min in the MILS−FLF group (p = 0.682). The mean blood loss was 301 ml in the MILS + FLF group vs. 318 ml in the MILS−FLF group (p = 0.859). Overall morbidity was 3.3% in the MILS + FLF group vs. 18.8% in the MILS−FLF group (p = 0.114). One patient in the MILS−FLF group (overall 2.2%), who underwent robotic liver surgery, developed bile leakage, but this did not occur in the MILS + FLF group. Covering the resection surface of the liver after minor minimally invasive liver resection with an FLF is a simple and cost-effective technique that does not prolong surgical time or negatively affect other perioperative parameters. In fact, it is a safe add-on step during MILS that may reduce postoperative morbidity. Further studies with larger cohorts will be needed to substantiate our proof of concept and results.

## Introduction

Minimally invasive liver surgery is becoming increasingly accepted as a safe and feasible procedure^1,2^. Laparoscopic liver resection shows better short-term outcomes than open liver surgery. Although robotic liver surgery is inferior to conventional laparoscopic surgery due to its high costs, robotic surgery also has certain benefits, such as a higher degree of freedom of movement and stable surgeon-controlled visualization^1,3,4^. In complex cases, robotic liver resection has shown significantly less blood loss than conventional laparoscopic resections^5^. A recent meta-analysis by Machairas et al. revealed that robotic liver surgery had significantly lower rates of overall morbidity and shorter lengths of postoperative stay but longer operating times than open liver surgery^6^. A high body mass index (BMI) was found to not be a barrier against robotic liver surgery^7^. Interestingly, Sultana et al. found that second-generation surgeons have a shorter learning curve for laparoscopic minor liver resection than pioneer surgeons^8^. Additionally, elderly patients can benefit from laparoscopic liver resection since it is considered safe and feasible in this population^9^.

In the treatment of hepatocellular carcinoma (HCC), both laparoscopic and open liver resection are comparable in terms of their overall and disease-free survival rates^10,11^. Wu et al. found open liver resection to be an independent risk factor for posthepatectomy liver failure (PHLF), while laparoscopic surgery was not^10^. However, conversion from laparoscopic to open liver resection for patients with hepatocellular carcinoma had a significantly negative impact on overall survival^12^.

The complication rate (36%) after liver surgery is high^13^. Posthepatectomy liver failure, bile leakage, posthepatectomy hemorrhage and vascular occlusion are specific complications of liver resection^14^. Apart from vascular occlusion, these complications are defined by the International Study Group of Liver Surgery (ISGLS). The ISGLS divides these complications into three grades: A, B, and C^15^. The rates of bile leakage and posthepatectomy liver failure amount to 8% and 5%, respectively^13^. A meta-analysis indicated an overall 30-day mortality of 1.4% after liver surgery: 0.7% after laparoscopic surgery and 1.6% after open liver surgery^16^. An Italian multicenter study showed overall morbidity and mortality rates of 22.8% and 0.2%, respectively, after minimally invasive liver surgery^17^. In a study with a large patient cohort consisting of 1,152 open hepatectomies for colorectal metastasis, the overall and surgical morbidity were 24.6% and 3.1%, respectively^18^. The overall morbidity in robotic liver surgery can reach up to 27%^19–21^.

Even if morbidity can be decreased in minimally invasive procedures, the mastery and management of complications remains a very important aspect of liver surgery.

In 2006, Ozmen et al. described a falciform ligament flap for filling the residual hole after resection for hydatid liver disease as a simple, secure and effective surgical technique to accelerate the elimination of the residual cystic cavity^22^. Currently, the falciform ligament flap is used in pancreatic surgery to reduce postoperative morbidity^23–25^. Moreover, the falciform ligament flap can be applied for laparoscopic repair of the crural defect caused by paraesophageal hernia^26,27^.

However, the use of the falciform ligament flap technique in laparoscopic or robotic-assisted liver surgery has not been described thus far. The aim of our work is to present a new approach to this surgical procedure that might help to prevent postoperative complications after minimally invasive liver surgery.

## Materials and methods

### Patients

Patients who underwent minor minimally invasive liver resection (< 3 segments) between 2011 and 2019 for benign or malignant liver tumors were selected from the Magdeburg registry of minimally invasive liver surgery (MD-MILS). Either full robotic-assisted or laparoscopic procedures were accepted. No hybrid or hand-port-assisted techniques were considered. Patients who underwent liver cyst deroofing were excluded from the study. After establishing the size and perfusion of the falciform ligament for the creation of a flap, the falciform ligament flap technique was implemented for sealing the resection surface.

We identified 46 patients in our registry according to the selection criteria. The patient cohort was divided into two groups: the first group consisted of 30 patients who underwent minimally invasive liver surgery (MILS) with a falciform ligament flap (FLF), including 16 laparoscopic and 14 robotic minor resections (MILS + FLF); the second group consisted of 16 patients who underwent MILS without FLF, including 8 laparoscopic and 8 robotic minor resections (MILS−FLF).

### Statistical analysis

We analyzed perioperative parameters and patient characteristics between the MILS + FLF and MILS−FLF groups.

The patient data were collected retrospectively. Data analysis was carried out using IBM SPSS Statistics for Windows, Version 26 (IBM Corp., Armonk, N.Y., USA).

We used cross tables for the descriptive analysis of categorial variables. The data of these variables are presented as the numbers and percentages of patients. We applied the chi-square test or Fisher's exact test to determine significant differences among these data. The independent samples t-test was applied for the continuous variables, whose data are presented using the means and standard deviations (SDs). Statistical significance was considered at a p-value of < 0.05.

### Anatomy of the falciform ligament

The falciform ligament is a connective tissue structure covered with parietal peritoneum. It is located between the ventral abdominal wall and liver and extends from the diaphragm at the proximal point to the liver at the caudal point as a round ligament. Due to its embryologic origins, it contains the remnants of the umbilical vein. The proximal point ends at the transition to the diaphragm in the left and right coronary ligaments. Clinically, the falciform ligament contributes to the assessment of orientation during abdominal surgery^28^. The blood supply to the falciform ligament is provided by the left inferior phrenic artery and middle segment artery of the liver, while venous drainage occurs through the left inferior phrenic vein^28–30^.

A falciform ligament artery can be detected during laparotomy in approximately two-thirds of patients^31^. It can be determined radiologically in up to 95% of cases^31,32^.

### Surgical technique for creating a falciform ligament flap in minimally invasive liver surgery

**Figure 1:**
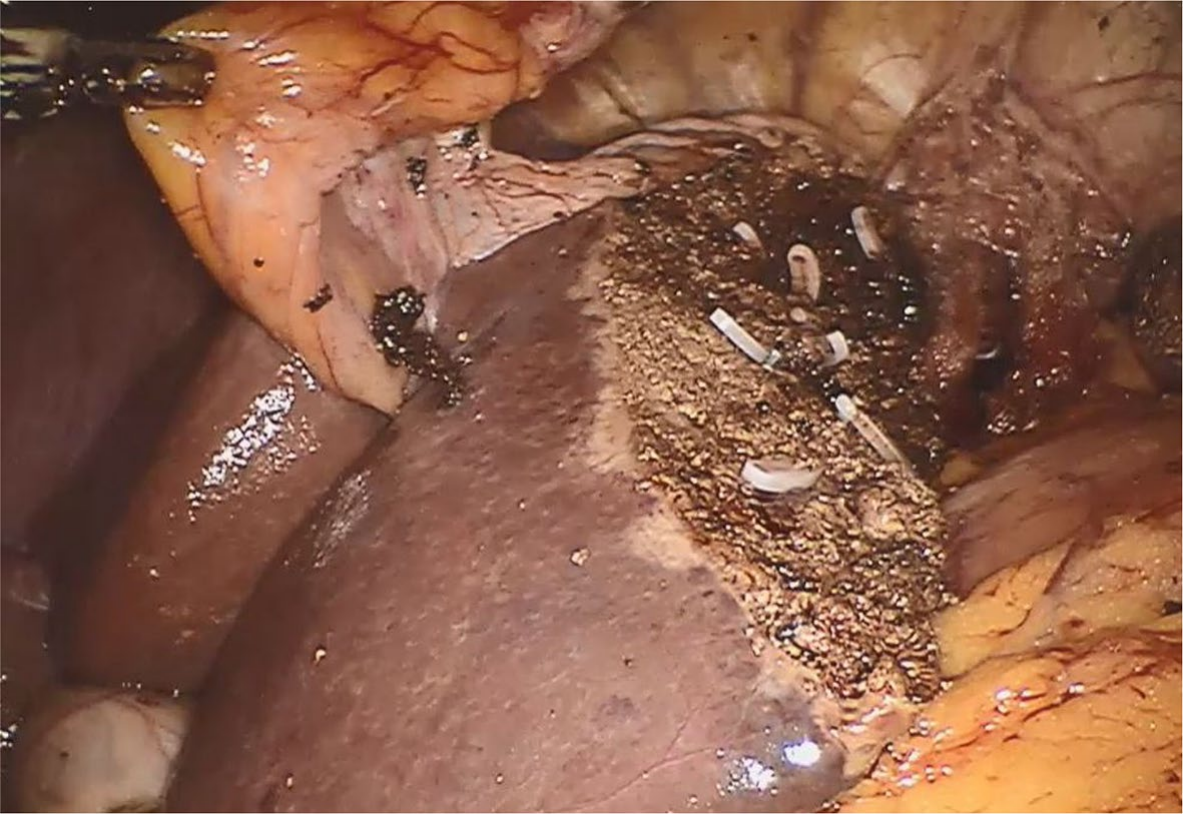
Resection surface of the liver after robotic removal of the left lateral segments and prepared falciform ligament flap.

**Figure 2:**
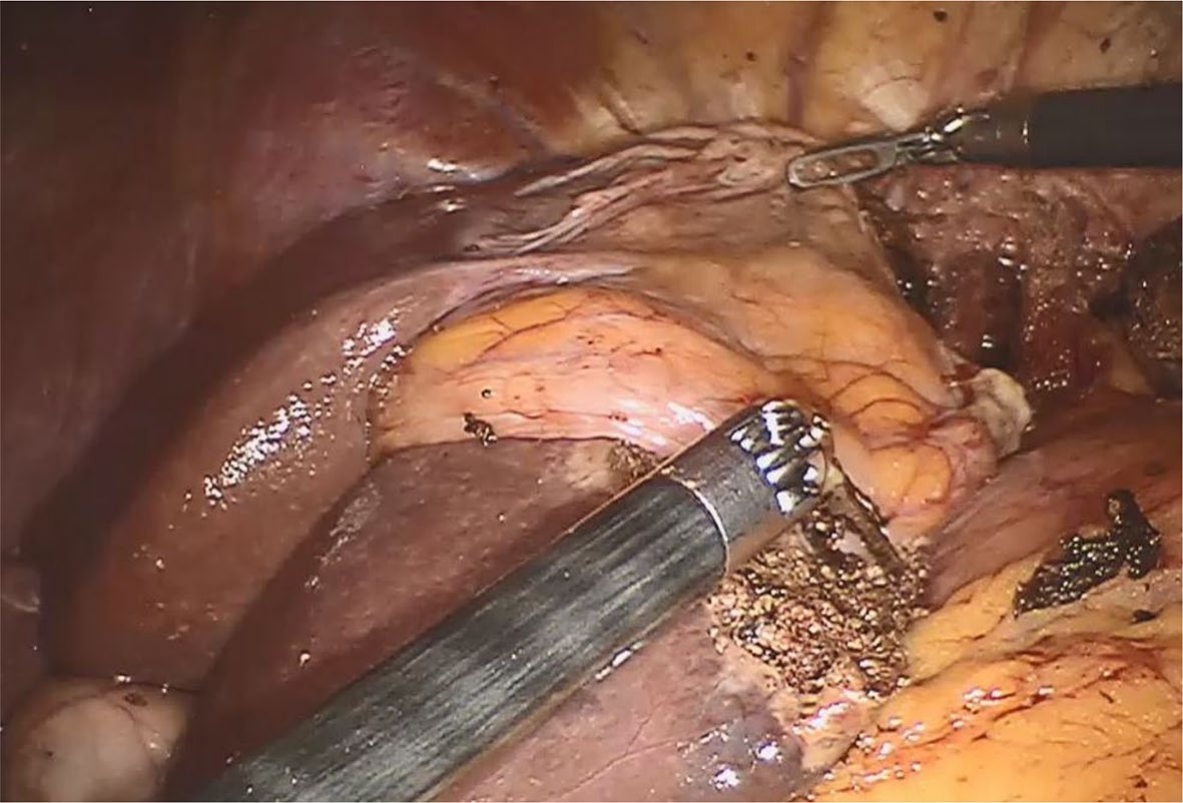
Covering of the hepatic resection surface with the mobilized falciform ligament flap.

**Figure 3:**
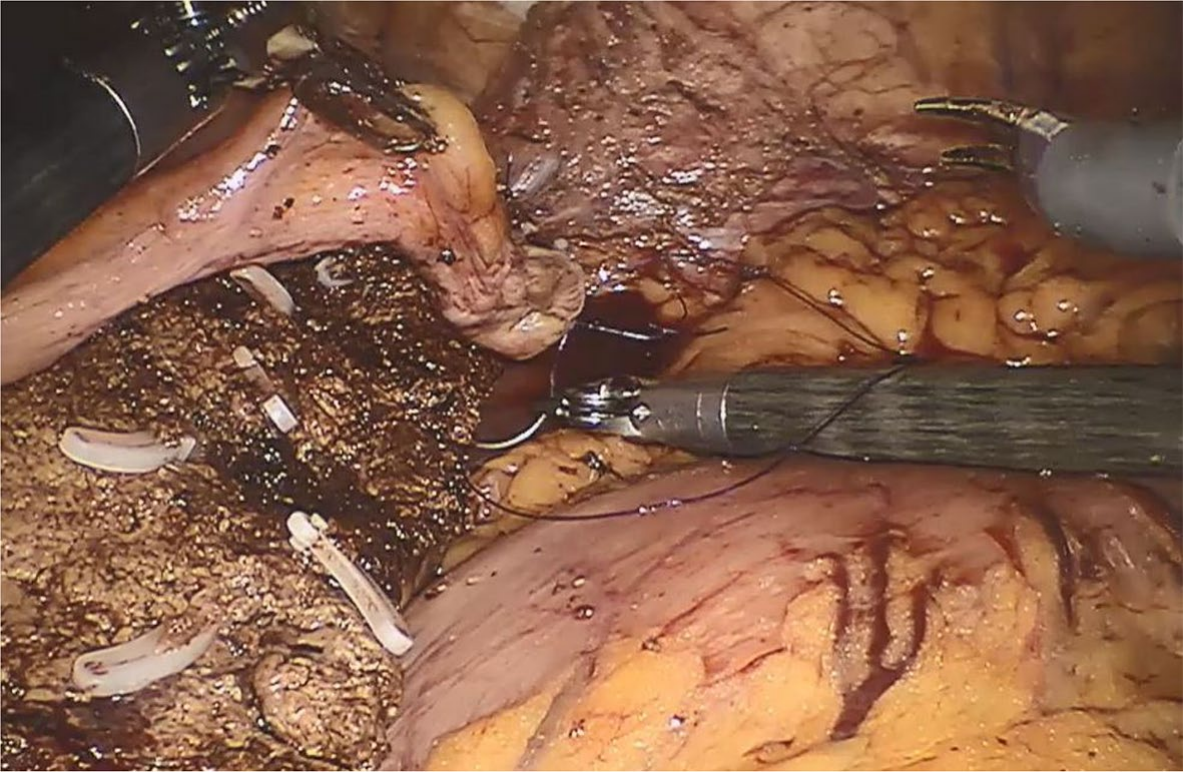
Robotic-assisted suture of the ventral edge of the falciform ligament flap on the dorsal margin of the resection surface of the liver.

**Figure 4:**
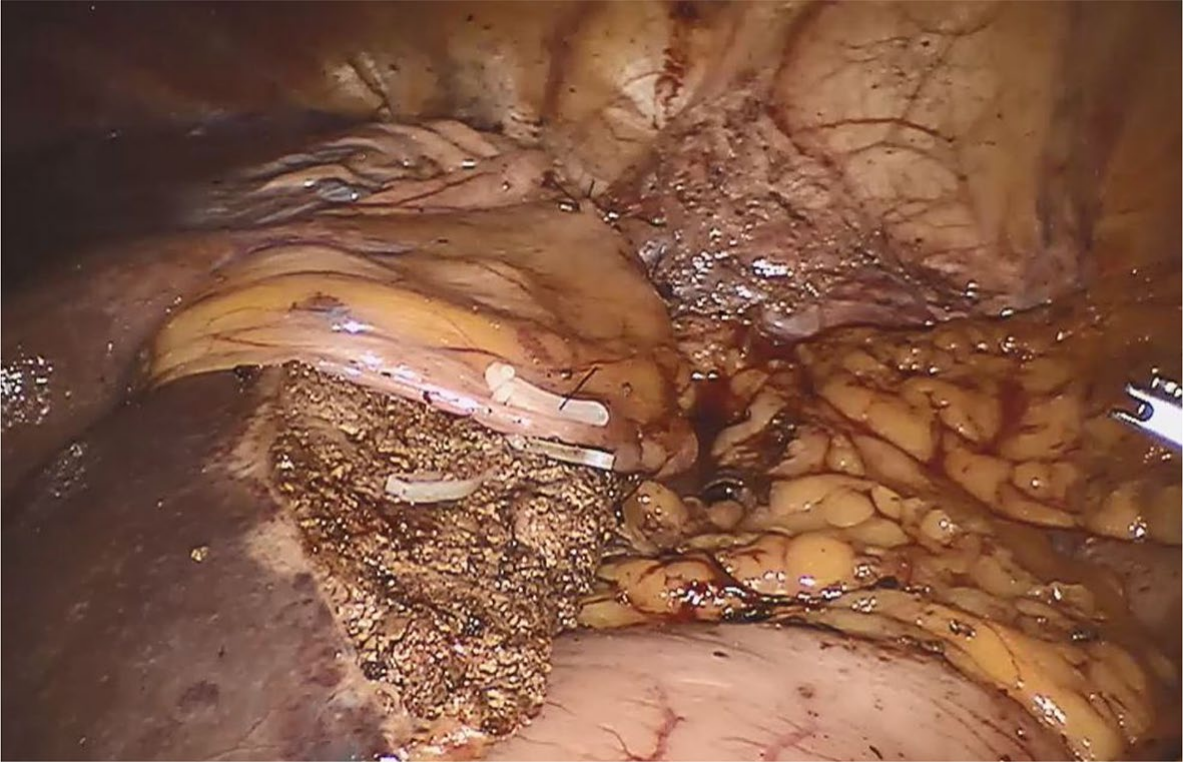
Finished falciform ligament flap covering the main area of the resection surface of the liver.

The principal surgical method for creating a falciform ligament flap is identical in laparoscopic and robotic-assisted liver surgery. After placement of the trocars, establishment of pneumoperitoneum and inspection of the situs, the falciform ligament is dissected along the border of the ventral abdominal wall. Here, a sealing device can prove useful. The separation is performed beginning from the navel and continues to the round ligament of the liver up to the lower wall of the diaphragm. In this way, a pedicled falciform ligament flap is created. The dorsal side of the falciform ligament flap is attached to the liver, and the proximal part may be partially adhered to the diaphragm. The attachment to the diaphragm should be dissected as little as possible because it is not irrelevant for the vascularization of the flap. In cases where the ligament is severed from the lower diaphragmatic wall, there should still be sufficient perfusion of the flap because vascularization of the ligament is variable. Depending on which lobe of the liver is undergoing resection, the separated falciform ligament, which is completely free on the ventral side, can be turned to the hepatic resection surface. After liver resection, the ventral free end of the flap is sutured onto the dorsal margin of the resection surface. As a result, the main area of the resection surface is now covered with the flap (Figs. 1, 2, 3, 4).

### Ethical pproval

All the protocols followed were approved by the ethics committee of the University Hospital Magdeburg. Informed consent in concordance with the Helsinki declaration regarding patient care was obtained from all patients.

## Results

### Patient demographics, surgery and liver tumors

Twenty-eight patients (60.9%) in our cohort were male. The overall mean age was 56.8 years (SD 16.8). The mean operating time was 244.2 min (SD 111.4). The patients spent 7.5 days (SD 4.9) in the hospital. The mean blood loss was 307 ml (SD 306.4).

Left lateral liver resection was the most common procedure, performed in 24 patients (52.2%). Anatomical liver segment resection was performed twelve times (26.1%). The remaining patients underwent various other liver resections, including < 3 liver segments (Table 1).

**Table 1:** Procedures and liver tumor pathology of patients who underwent minor minimally invasive liver surgery (MILS) with or without a falciform ligament flap (FLF) for sealing the resection surface.

	MILS + FLF n (%)	MILS—FLF n (%)	Total n (%)
**Type of liver resection (p = 0.611)**			
Left lateral LR	17 (56.7)	7 (43.8)	24 (52.2)
Anatomical liver segment resection	7 (23.3)	5 (31.3)	12 (26.1)
Bisegmentectomy	2 (6.7)	3 (18.8)	5 (10.9)
Atypical one-segment resection	3 (10.0)	1 (6.3)	4 (8.7)
Anatomic resection of two liver segments	1 (3.3)	0 (0.0)	1 (2.2)
Total	30 (100.0)	16 (100.0)	46 (100.0)
**Type of liver lesion (p = 0.305)**			
HCC	9 (30.0)	2 (12.5)	11 (23.9)
Colorectal metastases	8 (26.7)	3 (18.8)	11 (23.9)
FNH	5 (16.7)	3 (18.8)	8 (17.4)
CCA	1 (3.3)	2 (12.5)	3 (6.5)
Liver cyst	1 (3.3)	2 (12.5)	3 (6.5)
Hepatic adenoma	2 (6.7)	1 (6.3)	3 (6.5)
Uveal melanoma metastasis	2 (6.7)	0 (0.0)	2 (4.3)
Liver hemangioma	1 (3.3)	0 (0.0)	1 (2.2)
GIST metastasis	1 (3.3)	0 (0.0)	1 (2.2)
Metastasis of yolk sac tumor	0 (0.0)	1 (6.3)	1 (2.2)
Metastasis of ovarian carcinoma	0 (0.0)	1 (6.3)	1 (2.2)
Metastasis of hypopharyngeal carcinoma	0 (0.0)	1 (6.3)	1 (2.2)
Total	30 (100.0)	16 (100.0)	46 (100.0)

*CCA* cholangiocellular carcinoma, *FLF* falciform ligament flap, *FNH* focal nodular hyperplasia, *GIST* gastrointestinal stromal tumor, *HCC* hepatocellular carcinoma, *LR* liver resection, *MILS* minimally invasive liver surgery.

The final diagnosis was based on histopathological examination of the resected specimens. More than two-thirds of patients (67.4%) had a malignant tumor. Benign liver lesions were detected in fifteen patients (32.6%). The most common diagnoses were hepatocellular carcinoma (HCC) and colorectal liver metastases in eleven patients (23.9%). The second most common diagnosis was focal nodular hyperplasia in eight patients (17.4%). Less common malignant diagnoses were cholangiocellular carcinoma (CCA), uveal melanoma metastases, gastrointestinal stromal tumor (GIST), yolk sac tumor or ovarian and hypopharyngeal carcinoma metastases. In terms of other benign lesions, findings included liver cyst, hepatic adenoma and liver hemangioma (Table 1).

### Minimally invasive surgery with or without a falciform ligament flap

We recorded four postoperative complications (8.7%). One (3.3%) was in the group that received a falciform ligament flap (MILS + FLF group). This patient developed an enterocutaneous fistula from the small intestine postoperatively (Clavien-Dindo grade II), which did not need surgery. The other three patients (18.8%) with complications were in the group without an FLF. One patient in this group developed acute renal failure (Clavien-Dindo grade I) after surgery. The second patient developed a perforated duodenal ulcer after laparoscopic resection of hepatocellular carcinoma (HCC). This patient died due to peritonitis (Clavien-Dindo grade V). The third patient developed a postoperative bile leak (Clavien-Dindo grade IIIa). This complication was the only specific liver surgery-related complication (6.3%, overall 2.2%) in our study^15^. In the MILS + FLF group, no specific liver surgery-related complications were detected (Table 2).

**Table 2:** Demographics and perioperative outcomes of patients who underwent minor minimally invasive liver surgery (MILS) with or without a falciform ligament flap (FLF) for sealing the resection surface.

	MILS + FLF n (%) or mean (SD)	MILS—FLF n (%) or mean (SD)	p-value
Total	30	16	
**Sex**			
Male	18 (60.0)	10 (62.5)	0.869
Female	12 (40.0)	6 (37.5)	
Age; years	58.2 (15.2)	54.2 (19.7)	0.451
Operating time; minutes	249.2 (106.6)	234.8 (122.9)	0.682
LOPS; days	6.5 (2.5)	9.3 (7.5)	0.177
Blood loss; ml	301.0 (284.0)	318.1 (354.2)	0.859
Overall morbidity	1 (3.3)	3 (18.8)	0.114
Liver surgery related morbidity	0 (0.0)	1 (6.3)	0.348
Previous abdominal surgery	10 (33.3)	8 (50.0)	0.270
**Liver malignancy**			
Yes	21 (70.0)	10 (62.5)	0.605
No	9 (30.0)	6 (37.5)	

*FLF* falciform ligament flap, *LOPS* length of postoperative stay, *MILS* minimally invasive liver surgery, *SD* standard deviation.

Eighteen patients (39.1%) had undergone previous abdominal surgery and had relevant intra-abdominal adhesions. Ten of them (33.3%) were in the MILS + FLF group, and eight (50%) were in the MILS−FLF group.

Thirty-one patients (67.4%) had a malignant tumor. Twenty-one patients (70%) were in the MILS + FLF group, and ten (62.5%) were in the MILS−FLF group. In the remaining 15 patients (32.6%), different benign liver lesions were detected (Table 2). Based on the histopathological examination, we evaluated the surgical margin status in the malignant cases. Only one patient (10%, overall 3.2%) in the MILS−FLF group, which underwent atypical segment resection due to cholangiocarcinoma, showed a microscopic positive resection margin.

## Discussion

Our mean operating time (244.2 min) was comparable with other studies, which have reported mean operating times ranging from 146 to 261 min^2,7,33,34^. Adding an FLF at the end of the procedure did not substantially increase operative times.

The mean hospitalization time in our study was 7.5 days. This included 6.5 days for the MILS + FLF group and 9.3 days for the MILS−FLF group. In the non-FLF group, we had three patients with complications with consecutive prolonged LOPS. This led to a distortion of our results. Other studies showed a shorter LOPS with a mean hospitalization stay of 3–5 days^7,33,35^. First, Rebibo et al. presented a pilot study with minor laparoscopic liver resections as a day-case surgery without overnight hospitalization^36^. Nevertheless, this short hospitalization is not possible in Germany given its reimbursement system.

The overall mean blood loss was 307 ml in our study. Some authors reported mean blood losses between 111 and 175 ml for robotic minor liver surgery^2,7,34^. Our cohort consisted of laparoscopic and robotic-assisted cases, and in both groups, we had a substantial number of patients with adhesions from previous surgery. Furthermore, atypical segment resections were included. These findings may explain the differences between our findings and those of previous studies.

The overall morbidity was 8.7% in our study. Liver surgery-related morbidity was 2.2%, with only one patient developing a specific postoperative complication after robotic liver surgery. This patient did not receive a falciform ligament flap. The patients with falciform ligament flaps did not suffer any specific liver surgery-related postoperative complications. Melstrom et al. reported a complication rate of 9% among 87 patients who underwent robotic liver surgery^2^. Another study noted five patients (13%) with postoperative complications after robotic liver surgery^7^. A meta-analysis showed 15.59% overall morbidity for laparoscopic liver surgery in hepatocellular carcinoma patients^37^.

Recently, a meta-analysis showed that the use of topical hemostatic agents in liver surgery significantly decreased the time to hemostasis. However, it does not influence blood transfusion, the development of postoperative collections or bile leak^38^. The use of FLFs for sealing the resection surface can be a more efficient and cheaper alternative to the use of hemostatic agents for achieving lower rates of morbidity.

The MILS + FLF group showed shorter LOPS, less blood loss and lower rates of morbidity than the MILS−FLF group. However, the difference was not statistically significant. This may be due to the small number of patients in our cohorts. The primary aim of the study was to show the safety and feasibility of the FLF resection surface sealing method, which could be achieved with satisfactory results. Furthermore, we were able to show the noninferiority of this technique compared with the routine procedure. To demonstrate the superiority of the FLF sealing procedure, studies with more patients, ideally as part of a randomized controlled trial, are required.

## Conclusion

Hence, we showed that sealing the resection surface with an FLF after minimally invasive liver surgery did not have any negative impact on operating time or LOPS and may reduce postoperative complication rates. It is a cheap, feasible and simple procedure that can easily be added to minimally invasive liver resections and may possibly be a good alternative for topical hemostatic agents. Our first results from using this technique are promising. To date, the falciform ligament flap/patch technique has been successfully used in open liver surgery and pancreatic and hiatal hernia surgery. Further randomized studies will be needed to substantiate our proof of concept and the preliminary results.
